# Draft genome sequence of *Selenomonas sputigena* strain ATCC 33150 isolated from human mouth

**DOI:** 10.1128/mra.00827-25

**Published:** 2025-11-06

**Authors:** Taojun Wang, Colin G. Hawkes, Jason M. Ridlon, Daniel P. Miller

**Affiliations:** 1 Department of Animal Sciences, University of Illinois Urbana-Champaign 14589, Urbana, Illinois, USA; 2 Carl R. Woese Institute for Genomic Biology 124518, Urbana, Illinois, USA; 3 Philips Institute for Oral Health Research, Virginia Commonwealth University, School of Dentistry 6889, Richmond, Virginia, USA; 4 Department of Microbiology and Immunology, School of Medicine, Virginia Commonwealth University 6889, Richmond, Virginia, USA; 5 Division of Nutritional Sciences, University of Illinois at Urbana-Champaign 14589, Urbana, Illinois, USA; 6 Cancer Center at Illinois, University of Illinois at Urbana-Champaign 14589, Urbana, Illinois, USA; 7 Center for Advanced Study, University of Illinois Urbana-Champaign 14589, Urbana, Illinois, USA; Nanchang University, Nanchang, Jiangxi, China

**Keywords:** *Selenomonas*, periodontitis, caries

## Abstract

*Selenomonas sputigena* is an anaerobic, motile, crescent-shaped, gram-negative bacterium found in the human oral cavity that is associated with dental caries and periodontitis. Strain ATCC 33150 was replaced as the type strain for *S. sputigena* and was described as atypical, as it fermented arabinose and displayed a unique pattern by polyacrylamide electrophoresis compared to other isolates. The genome of the atypical strain, ATCC 33150, is 2,601,282 base pairs in length, comprising 2,342 protein-coding genes, and a 54.68% GC content.

## ANNOUNCEMENT

*Selenomonas sputigena* has historically been associated with periodontitis and has recently been implicated in exacerbating early childhood caries (1–6). This makes *S. sputigena* a unique oral bacterium that contributes to diseases above and below the gum line. The ATCC 33150 (VPI 10068) strain was previously designated as the type strain of *S. sputigena*; however, a petition to replace this strain was submitted, as it uniquely fermented arabinose and was distinct from six other strains of *S. sputigena* by DNA homology (7). In 1992, ATCC 33150 was officially replaced as the type strain by ATCC 35185 (8). Here, we report the draft genome sequence for this atypical *S. sputigena* strain, ATCC 33150.

*S. sputigena* ATCC 33150 was purchased from the American Type Culture Collection (ATCC) and grown anaerobically (90% N_2_, 5% H_2_, and 5% CO_2_) in tryptone-yeast extract-gelatin-volatile fatty acids-serum broth at 37°C in an anaerobic chamber (Coy Laboratories). Culture purity was established by observation under dark-field microscopy (9). High molecular weight (HMW) DNA extraction, library preparation, and sequencing were conducted at the Roy J. Carver Biotechnology Center at the University of Illinois at Urbana-Champaign. Cell pellets were washed and resuspended with 200 µL phosphate-buffered saline (Corning, VA). Cell lysis was done with 5 µL Metapolyzyme (Sigma, MO) and a 2 h incubation at 37°C. Additional lysis was done with the MagAttract HMW DNA Kit (Qiagen, MD). Chloroform was added, and the samples were centrifuged at 10,000× g for 2 min to isolate the supernatant containing the DNA. DNA purification was subsequently carried out using the MagMAX Plant DNA Isolation Kit (Thermo Fisher Scientific, MA), with buffers prepared according to the manufacturer’s instructions. DNA was eluted in 50  µL of elution buffer and quantified using Qubit (Thermo Fisher Scientific), and fragment sizes were evaluated in the Femto Pulse system (Agilent, CA). The HMW DNA was sheared with a Megaruptor 3 to an average fragment length of 10 kb. Sheared DNA was converted to a library with the SMRTBell Express Template Prep kit 3.0. The library was sequenced on a shared SMRT cell 8 M on a PacBio Revio using the circular consensus sequencing (CCS) sequencing mode and a 30 h movie time. CCS analysis was done in an instrument with SMRTLink V13.1 using the following parameters: ccs --min-passes 3 --min-rq 0.99.

Read quality was evaluated using FastQC v0.11.8 (10). SeqKit v2.0.0 (11) was used to calculate the statistics of the sequencing files. Flye v2.9 (12) was used to assemble the reads. Assembly quality was evaluated using QUAST v5.0.2 (13). Completeness and contamination were calculated with CheckM v1.1.9 (14). Annotations were performed using the Prokaryotic Genome Annotation Pipeline (PGAP) version 6.10, upon submission to NCBI (15). For all software, default parameters were utilized unless otherwise noted. A total of 393,509 reads were sequenced, with 100 million base pairs (bp) used for the assembly. A single circular contig assembly was generated (N50 = 2,601,282). The genome coverage was 713. The total length of the contig is 2,601,282 bp, with a GC content of 54.68%. The assembled genome was 99.07% complete with 0.63% contamination. ATCC 33150 contains 2,342 protein-coding genes, 12 rRNA genes, 53 tRNA genes, and 5 ncRNA genes. The average nucleotide identity of ATCC 33150 to the *S. sputigena* reference genome (ATCC 35185) was determined to be 80% using the Anvi’O workflow (16), strongly suggesting ATCC 33150 represents a species distinct from *S. sputigena*.

## Data Availability

Project data are available at NCBI Genome Assembly Accession Number CP194411.1, BioProject PRJNA1272640, and BioSample SAMN48918871.
